# Drug repurposing for coronavirus (SARS-CoV-2) based on gene co-expression network analysis

**DOI:** 10.1038/s41598-021-01410-3

**Published:** 2021-11-08

**Authors:** Habib MotieGhader, Esmaeil Safavi, Ali Rezapour, Fatemeh Firouzi Amoodizaj, Roya asl Iranifam

**Affiliations:** 1 Department of Basic Sciences, Biotechnology Research Center, Tabriz Branch, Islamic Azad University, Tabriz, Iran; 2 Department of Biology, Tabriz Branch, Islamic Azad University, Tabriz, Iran; 3 Department of Basic Sciences, Faculty of Veterinary Medicine, Tabriz Branch, Islamic Azad University, Tabriz, Iran; 4 Department of Animal Science, Faculty of Agriculture, Tabriz Branch, Islamic Azad University, Tabriz, Iran

**Keywords:** Computational models, Gene ontology, Microarrays, Drug screening

## Abstract

Severe acute respiratory syndrome (SARS) is a highly contagious viral respiratory illness. This illness is spurred on by a coronavirus known as SARS-associated coronavirus (SARS-CoV). SARS was first detected in Asia in late February 2003. The genome of this virus is very similar to the SARS-CoV-2. Therefore, the study of SARS-CoV disease and the identification of effective drugs to treat this disease can be new clues for the treatment of SARS-Cov-2. This study aimed to discover novel potential drugs for SARS-CoV disease in order to treating SARS-Cov-2 disease based on a novel systems biology approach. To this end, gene co-expression network analysis was applied. First, the gene co-expression network was reconstructed for 1441 genes, and then two gene modules were discovered as significant modules. Next, a list of miRNAs and transcription factors that target gene co-expression modules' genes were gathered from the valid databases, and two sub-networks formed of transcription factors and miRNAs were established. Afterward, the list of the drugs targeting obtained sub-networks' genes was retrieved from the DGIDb database, and two drug-gene and drug-TF interaction networks were reconstructed. Finally, after conducting different network analyses, we proposed five drugs, including *FLUOROURACIL*, *CISPLATIN*, *SIROLIMUS*, *CYCLOPHOSPHAMIDE*, and *METHYLDOPA,* as candidate drugs for SARS-CoV-2 coronavirus treatment. Moreover, ten miRNAs including *miR-193b, miR-192, miR-215, miR-34a, miR-16, miR-16, miR-92a, miR-30a, miR-7,* and *miR-26b* were found to be significant miRNAs in treating SARS-CoV-2 coronavirus.

## Introduction

Coronaviruses infect both animals and humans, causing intestinal and respiratory infections^1,2^. Severe acute respiratory syndrome (SARS) is a coronavirus-associated respiratory disease that was originally discovered in China in February 2003^3^. Ten years after SARS coronavirus, Middle East respiratory syndrome (MERS) coronavirus was broke out in Middle Eastern countries^2,4,5^. SARS-CoV and MERS uses angiotensin-converting enzyme 2 (ACE2) and dipeptidyl peptidase 4(DPP4) as a receptor, respectively^2^. Additionally, in the autumn of 2019, the coronavirus SARS-CoV-2 broke out in the Chinese city of Wuhan^4,6^. Given that, there is a high similarity between SARS-CoV and SARS-CoV-2, the spread speed of SARS-CoV-2 is faster than SARS-CoV^4^. SARS-CoV-2 like SARS-CoV utilize the host cell ACE2 receptor^4^. ACE2 is a membrane receptor on the surface of many cell types and tissues including the lungs, heart, blood vessels, kidney, liver, and gastrointestinal^2,4^. SARS-CoV, SARS-CoV-2, and MERS all have similar genetic characteristics, and SARS-CoV-2 is very similar to SARS-CoV^7^. Due to the high genetic similarity of SARS-CoV and SARS-CoV-2, the results of the study on SARS-CoV can be a clue to the treatment of SARS-CoV-2. Due to the disease's significance and high death rate, early detection and treatment are essential. The medicinal drugs used to treat coronavirus infections are only intended to be used temporarily. Besides, clinical trials on medications and vaccines that are efficient in curing diseases take quite a long time. Furthermore, handling SARS viruses in vivo is often challenging and risky. However, the knowledge obtained by sequencing their genes, proteins, or RNA is simple and easy to manage through artificial intelligence ^8^. Additionally, miRNA-mRNA data sources have progressed considerably as prospective techniques for gaining a better understanding of potential SARS-CoV therapies, allowing network science and computational systems biology to become feasible9. According to these data, many scientists seek to identify the involved host genes and proteins in diseases to find a new therapy.

Recent research has identified a set of antiviral genes, such as ISG15, IFIH1, MX1, OAS1-3, IRF7, IRF9, and STAT1 expressed by host cells, which could be used as a new therapeutic target against coronavirus due to their response to viral infection^10–18^. In addition to therapeutic gene targets, miRNAs can also be used to suppress the viral genome due to their ability to regulate gene expression. MiRNAs are small non-coding RNA molecules that prevent mRNAs from being translated^19,20^. Therefore, miRNA-based therapy could be proposed for SARS-CoV treatment^21,22^. On the other hand, the role of pathologic processes in miRNAs, such as inflammatory responses and viral infection, has been recently verified^21,23,24^.

Drug repurposing (DR) is a strategy for identifying new therapeutic uses for approved or investigational drugs^25,26^. This approach is also referred to as drug reprofiling, drug re‑tasking, drug repositioning, drug therapeutic and drug recyclining^26^. It is an efficient approach for the development or discovery of drug molecules with new therapeutic indications^25^. Generally, the process of drug repurposing consists of three steps^26^: 1. identification of a candidate molecule for a particular indication. 2. mechanistic evaluation of the drug effect in preclinical models. 3. evaluation of efficacy in phase clinical trials II. Of these three steps, step 1 is crucial, and it is here that modern approaches to hypothesising may be most useful^26^. These systematic approaches can be divided into experimental and computational approaches^26^. Some of the computational approaches are: *molecular docking*, *signature machiing*, *genetic association*, *network mapping*, *retrospective clinical analysis* and *novel data sources*^26^. As well as, among the experimental methods, *Binding assays to identify relevant target interactions* and *Phenotypic screening* approaches can be mentioned^26^.

Recently, different articles based on network approaches have been published for drug repurposing^27^. SAveRUNNER^28^ is a network-based drug repurposing algorithm, which predicts drug-disease assosiations using network-based similarity measure. This algorithm provided as a freely available R-code^29^. SAveRUNNER is also been used as a drug repurposing tool for amyotrophic lateral sclerosis (ALS) disease^30^. Pasquale and colleaqes^31^, examined three different network-based approaches and identified 399 repurposable drugs for COVID-19 using SAveRUNNER algorithm. Another algorithm based on artificial intelligence, network diffusion, and network proximity introduced as a drug repurposing method for SARS-CoV-2 disease^32^. We also recently introduced a protein–protein interaction network approach in order to propose candidate drugs to treatment of SARS-CoV-2 disease^21^.

Tasnimul and colleague, recently introduced a network-based method for identifying and repurposing drugs for the treatment of SARS-CoV-2 disease^33^. In this method, differentially expressed genes between Idiopathic pulmonary fibrosis (IPF) and SARS-CoV-2 samples were compared and finally, some IPF drugs were proposed as candidate drugs to treat SARS-CoV-2 disease. Yadi et al.^34^ proposed a novel network-based drug repurposing methodology based on human interactome and protein–protein interaction networks. This method quntify the interplay between the drug targets and HCoV–host interactome in the human protein–protein interaction network. In this study, 16 potential drugs was introduced to treat SARS-CoV-2 disease. In another study, Hangyu and colleaque^35^ developed a machine-learning -based method to predict virus-host interactions at both organism and protein levels for SARS-Cov-2 disease. In this method, a multi-layer virus-host interaction network was constructed. CoVex^36^, an interactive online platform for SARS-CoV-2, introduced by Sepideh and colleaque. This platform, integrates human protein–protein interactions, virus-human protein interactions and drug-target interactions. Zhihao and colleaque^37^ constructed a autophagy interaction network based on competitive endogenous RNA(ceRNA) in SARS-CoV-2 infection. In this study, hsa-miR-4772–5p, hsa-miR- 192–5p, hsa-miR-652–3p, hsa-miR- 192–5p, hsa-miR-340–3p, CCR2 and TP53INP2 introduced as potential biomarkers in predicting changes in mild SARS-CoV-2 infection. In comparison to the mentioned network-based methods, in our study, the gene co-expression network is used. In addition to the gene co-expression network, regulatory interactions including miRNA-Gene, TF-Gene, and TF-miRNA have also been used in our study. Moreover, drug-gene and drug-TF interaction networks have been studied and investigated in this study. As well as, some of the genes that regulate more miRNAs, are also introduced as effective miRNAs in SARS-Cov disease. Changes in the expression of these genes can affect the expression of target miRNAs.

The current research aimed to discover the genes and miRNAs involved in SARS-CoV disease and repurpose candidate drugs for this diease in order to treating SARS-CoV-2 coronavirus based on a co-expression network analysis. In this regard, this study used a co-expression network analysis to identify potential drugs for the treatment of SARS-CoV. The methodology we used in this study is an entirely novel method based on gene module identification.

**Figure 1:**
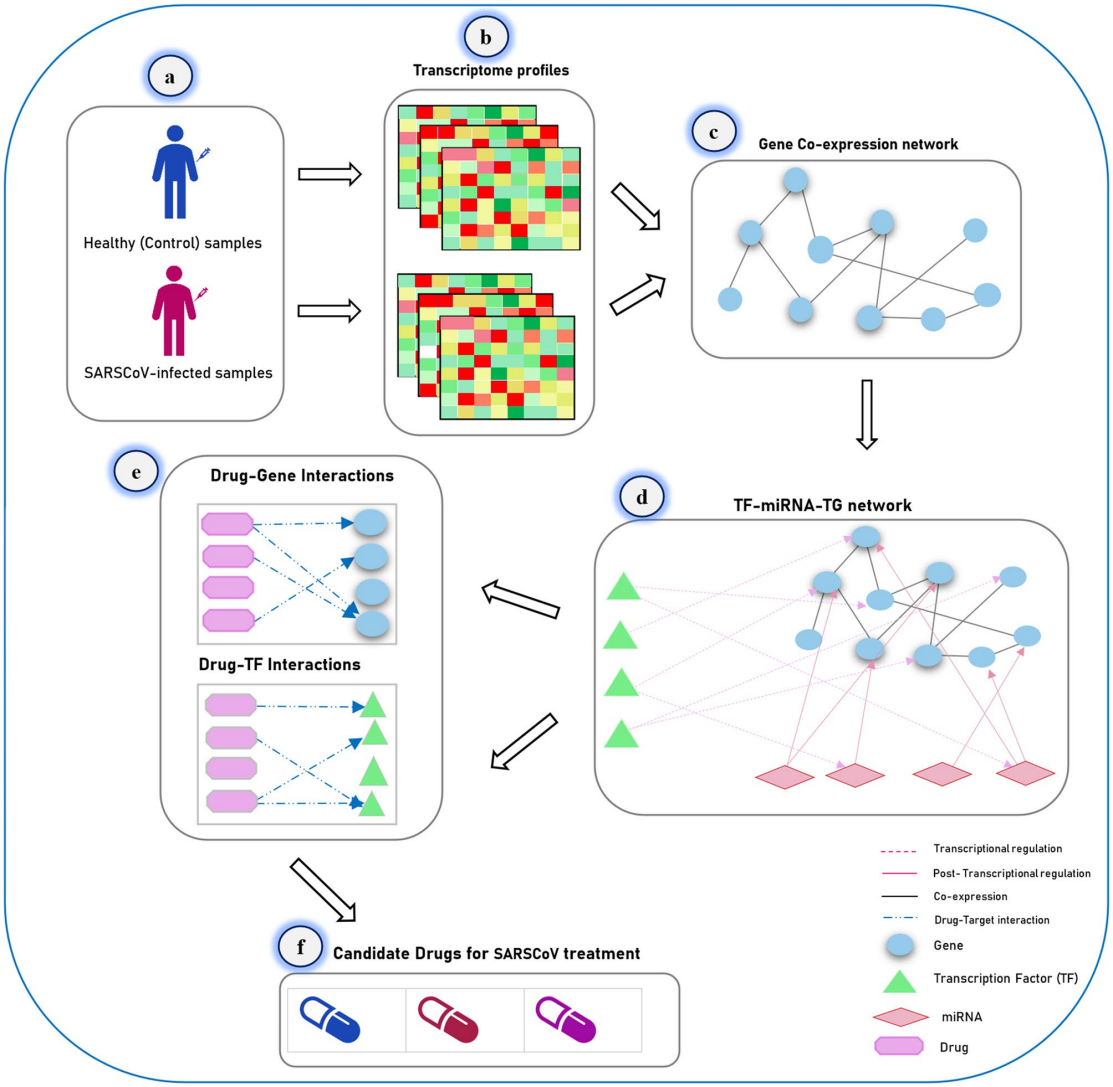
The overall workflow of the proposed method. In this method, a network-based approach is applied to drug repurposing for coronavirus disease treatment. (**a**) At first, a transcriptome profile for healthy (control) and SARS-CoV-infected samples were taken from the GEO database with the accession number GSE1739. (**b**) Then, after identifying differentially expressed genes in the control and disease groups, the gene co-expression network is reconstructed, and two significant gene modules are discovered from the co-expression network. (**c**) Next, for every gene module, the *TF-miRNA-TG* network is reconstructed independently. The information of TFs-miRNAs, TFs-TGs, and miRNAs-TGs regulations are taken from the *TransmiR*^42^, *TRRUST*^39^, and *miRWalk*^40^ databases, respectively. (**d**) Afterward*, Drug-gene* and *Drug-TF* networks are reconstructed for *TF-miRNA-TG* networks independently. (**e**) Finally, 19 drugs are proposed as candidate drugs for coronavirus treatment.

The technique first entails identifying a list of genes (human genes) expressed differentially in healthy and SARSCoV-infected samples. After obtaining differentially expressed genes between healthy and SARSCoV-infected samples, the co-expression network is reconstructed in STRING online tool^38^. Then, two significant gene modules are discovered from the gene co-expression network. Afterward, a list of miRNAs and transcription factor genes that have a regulatory impact on modules' genes are collected from a valid database (TRRUST v2^39^ and miRWalk v2^40^), and different network analyses are done on these biomolecules. Finally, the list of drugs that target modules' genes are gathered from the (DGIdb)^41^ database, and then two drug-gene interaction networks are reconstructed. The workflow diagram of this study is demonstrated in Fig. 1. As shown in this figure, the method's output is some candidate drugs for the treatment of SARS coronavirus. In this study, *FLUOROURACIL*, *CISPLATIN*, *SIROLIMUS*, *CYCLOPHOSPHAMIDE*, and *METHYLDOPA* are the key drugs reported for treating SARS-CoV-2 coronavirus. Moreover, *hsa-miR-193b, hsa-miR-192, hsa-miR-215, hsa-miR-34a, hsa-miR-16, hsa-miR-16, hsa-miR-92a, hsa-miR-30a, hsa-miR-7,* and *hsa-miR-26b* are candidate miRNAs, which are significant in the treatment of SARS-CoV-2 disease.

## Result

### Gene co-expression network analysis and gene modulation.

**Figure 2:**
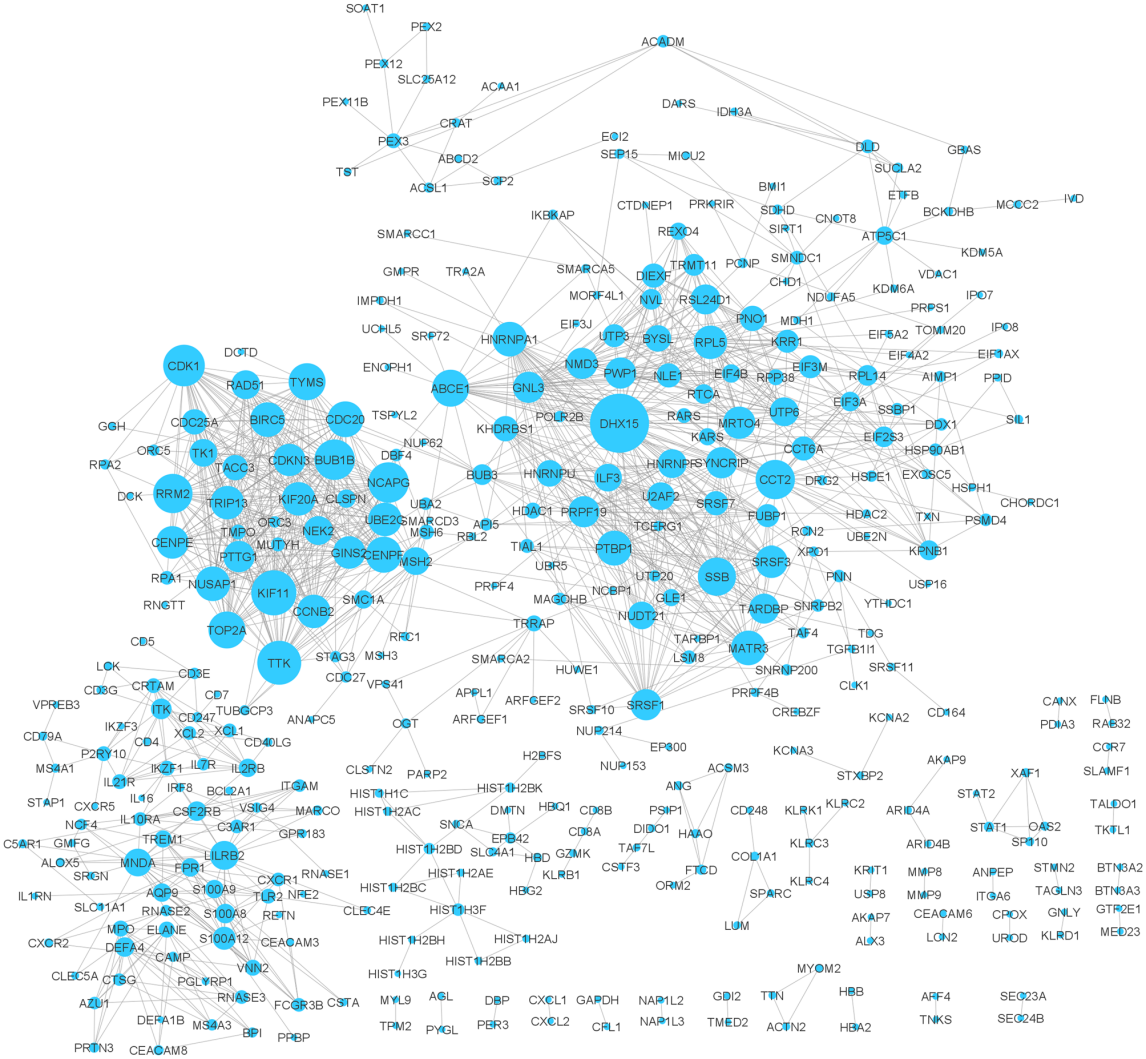
Gene co-expression network for the primary genes (disconnected genes were removed from the network). The size of the nodes indicates its degree. There are 391 nodes and 1273 edges in this network. DHX15 is the highest degree node in this network.

First 1441 differentially expressed genes between normal and SARS infected groups with *p-values* less than 0.05 were assumed as primary genes. Then, using this primary gene list, the gene co-expression network was reconstructed in the STRING database. In this network 1050 genes out of 1441 primary genes were disconnected. Therefore, these disconnected genes were removed from the network and a network with 391 genes was obtained. Figure 2 shows the co-expression network for these genes. Supplementary file S6 contains more information on the topological characteristics of this network.

After analyzing the network in Cytoscape software^43^, generally, 391 nodes and 1273 edges were observed in the reconstructed co-expression network. After clustering the gene co-expression network, we discovered two significant modules (Module A and Module B) in the co-expression network. The list of genes for these two modules is reported in Table 1.

**Table 1:** Gene names of modules A and B.

Module name	Gene names
Module A	TOP2A (24), TTK (24), NUSAP1(24), UBE2C(24), CENPF(24), KIF20A(24), CDK1(24), CDC20(24), BIRC5(24), TRIP13(23), NCAPG(24), GINS2(23), KIF11(24), CENPE(22), BUB1B(24), CCNB2(24), CDC25A(17), TACC3(17), RAD51(20), TYMS(22), CDKN3(22), RRM2(24), TK1(19), NEK2(19), PTTG1(22)
Module B	SRSF1(16), SYNCRIP(14), HNRNPU(16), DHX15(29), SSB(19), MATR3(17), GNL3(13), NUDT21(16), U2AF2(17), HNRNPF(17), SRSF7(15), SRSF3(17), KHDRBS1(14), FUBP1(13), PRPF19(14), BYSL(12), ILF3(17), NLE1(12), UTP6(12), RSL24D1(12), MRTO4(13), DIEXF(12), PTBP1(17), UTP3(12), PWP1(12), PNO1(12), KRR1(12), NMD3(12), HNRNPA1(17), TARDBP(17)

Numbers inside the parentheses represent the genes degree.

### Transcription factors, miRNAs, and target genes interaction network

At first two *TF-miRNA-TG* sub-networks named *TF-miRNA-TG*_A and *TF-miRNA-TG_B* were reconstructed for gene modules A and B, respectively. As demonstrated in previous section, two significant gene modules were considered for more analysis. To do this, the data of TFs-TGs, TFs-miRNAs, and miRNAs-TGs regulatory interactions were retrieved from the TRRUST, TransmiR, and miRWalk databases, respectively. These two sub-networks are shown in Figure S1 (See supplementary file S6). The information for TFs and TGs Interactions are *activation*, *repression,* and *unknown*. In addition, regulatory interactions information for TFs and TGs are: *activation*, *activation(feedback)*, *regulation*, *regulation(feedback)*, *autoregulatory negative feedback*, *loop(feedback)*, *repression*, *repression(feedback)*, *auto-regulatory feedback circuit,* and *activation (negative regulatory loop)*. This information for *TF-miRNA-TG_A* and *TF-miRNA-TG_B* sub-networks (see supplementary file S7) are reported in supplementary file S2. Moreover, the topological properties for these sub-networks was reported in supplementary file S6.

In order to analyze the TFs, which regulate more Genes and miRNAs in the *TF-miRNA-TG*_A and *TF-miRNA-TG*_B networks, the high degree TF nodes were selected and reported as significant TFs. To do this, among TF nodes in the *TF-miRNA-TG_A* and *TF-miRNA-TG_B* sub-networks, the TFs with a degree of 10 and higher were selected (Table 2). The number of TGs and target miRNAs for every TF are listed in Table 2 as well.

**Table 2:** High degree nodes of the TFs in the *TF-miRNA-TG_A and B* sub-networks.

Module name	TF	Number of TGs	Number of target miRNAs
*TF-miRNA-TG_A*	MYC	3	91
*TF-miRNA-TG_B*	TP53	5	89
	MYC	1	91
	NFKB1	1	67
	RELA	1	59
	STAT3	1	53
	MYCN	1	52
	SP1	4	38
	ESR1	1	30
	E2F3	1	20
	KLF4	1	18
	CTNNB1	1	16
	DNMT1	1	15
	NANOG	1	14
	TP73	2	12
	LEF1	1	12
	MYB	1	10
	FOXO3	1	9

In addition to the TFs, a list of miRNAs that regulate more genes in the *TF-miRNA-TG_A* and *TF-miRNA-TG_B* networks were selected and reported as key miRNAs. To this end, for every *TF-miRNA-TG* network, five miRNAs with high degrees were assumed. These miRNAs for *TF-miRNA-TG_A* were *hsa-miR-193b, hsa-miR-192, hsa-miR-215, hsa-miR-34a,* and *hsa-miR-16*. For *TF-miRNA-TG_B* network, the selected miRNAs were *hsa-miR-16, hsa-miR-92a, hsa-miR-30a, hsa-miR-7,* and *hsa-miR-26b.* Among these miRNAs*, hsa-miR-16* regulates more genes in both subnetworks. The list of these miRNAs, along with their degree and target genes, are reported in Table 3.

**Table 3:** High degree nodes of the miRNAs in the *TF-miRNA-TG_A and B* sub-networks.

miRNA	Degree	Target genes
*TF-miRNA-TG_A*		
*hsa-miR-193b*	13	RRM2, RAD51, CDC25A, CDK1, CDC20, NCAPG, KIF11, TYMS, UBE2C, TACC3, GINS2, TRIP13, BUB1B
*hsa-miR-192*	10	RAD51, CDC25A, CDC20, CENPF, CDKN3, KIF20A, TTK, TRIP13, BUB1B, CENPE
*hsa-miR-215*	9	RAD51, CDC20, CENPF, CDKN3, KIF20A, TTK, TRIP13, BUB1B, CENPE
*hsa-miR-34a*	7	RRM2, CDC25A, BIRC5, CDC20, NCAPG, KIF11, TYMS, hsa-miR-34a
*hsa-miR-16*	7	CDC25A, BIRC5, CDK1, CDC20, NCAPG, UBE2C, CENPF
*TF-miRNA-TG_B*		
*hsa-miR-16*	8	HNRNPF, HNRNPA1, SRSF1, NLE1, NMD3, DIEXF, UTP3, BYSL
*hsa-miR-92a*	7	HNRNPF, PTBP1, PNO1, NLE1, NMD3, KHDRBS1, U2AF2
*hsa-miR-30a*	6	HNRNPA1, HRNPU, SRSF7, UTP6, PTBP1, PRPF19
*hsa-miR-7*	4	HNRNPU, MATR3, SRSF1, hsa-miR-7, ILF3
*hsa-miR-26b*	4	MATR3, SYNCRIP, NMD3, GNL3

### Enrichment analysis of genes

Gene ontology was performed for the module A and B gene lists, separately. The results for module A gene list show that they significantly enriched in *mitotic cell cycle process, nuclear division,* and *mitotic nuclear division* biological processes. Moreover, the gene list of module B significantly enriched in *RNA processing*, *mRNA metabolic process,* and *RNA metabolic process* biological processes. Then, a pathway enrichment analysis was done for modules A and B gene lists separately. The results showed that the module A and B genes, significantly enriched in *Resolution of Sister Chromatid Cohesion* and *mRNA Splicing—Major Pathway* pathways, respectively. More details of the GO and pathway enrichment analyses are reported in supplementary file S3.

### Enrichment analysis of miRNAs

In order to check miRNA family for the *TF-miRNA-TG_A* and *TF-miRNA-TG_B* sub-networks, at first the list of miRNAs are imported into the TAM online tool. Then, the obtained result is reported in supplementary file S4. As reported in this file, both sub-networks significantly enriched in the *let-7* and *mir-17* families.

### Drug-Gene interaction network

**Figure 3:**
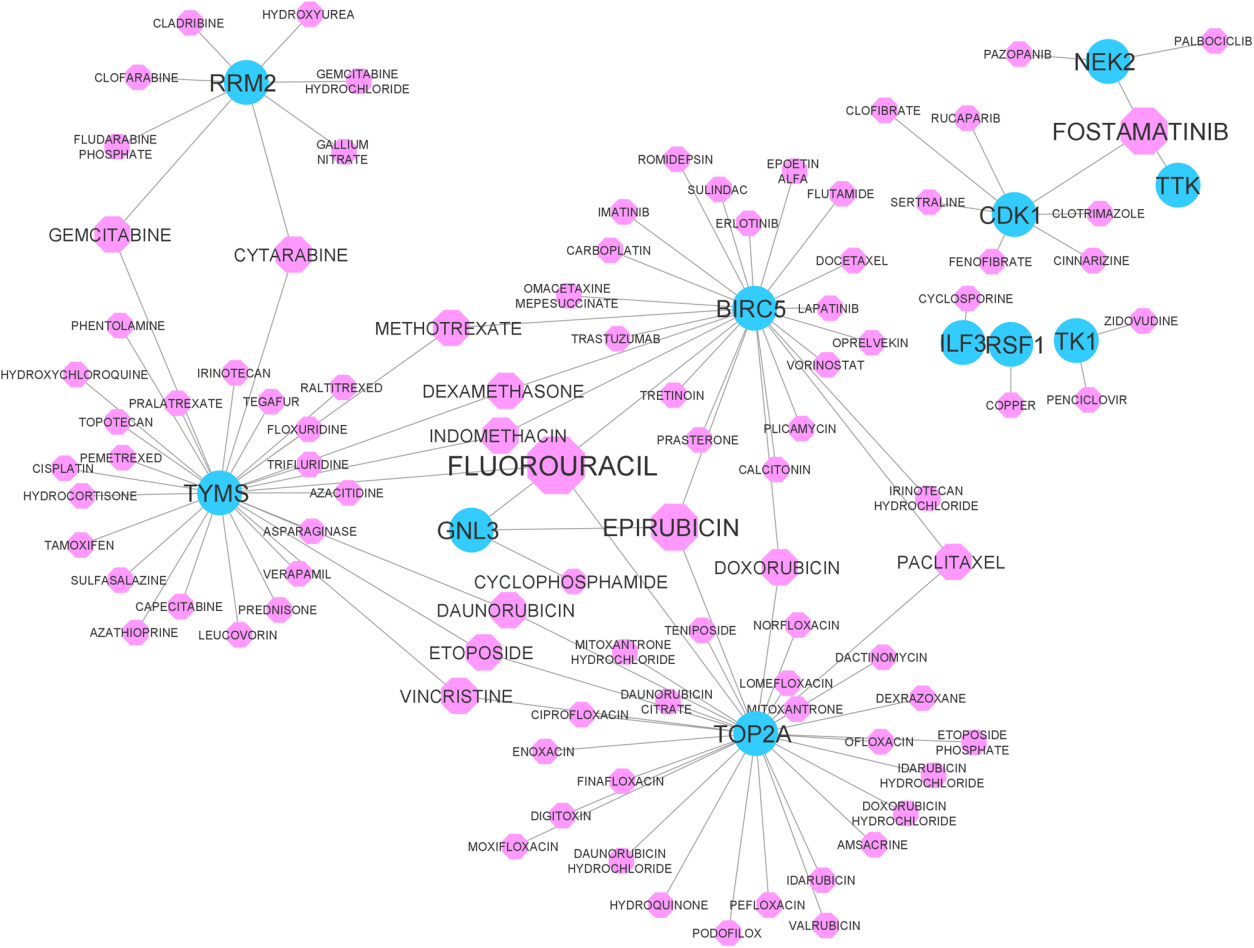
The drug-gene interaction network for *TF-miRNA-TG_A* and *TF-miRNA-TG_B* sub-networks. Blue circles show TGs, and pink octagons show drugs. The size of the octagon nodes indicates its degree. The high degree drug is FLUOROURACIL with 4 target genes.

After gathering drug-gene interactions for *TF-miRNA-TG_A* and *TF-miRNA-TG_B* genes, the drug-gene interaction network was reconstructed and is demonstrated in Fig. 3. As shown in this network, some drugs have a high degree, which means that these drugs target and regulate more genes. Therefore, high-degree drugs were selected and reported as significant, as they regulate more genes in *module_A* and *module_B.* In this regard, the drugs with a degree of 3 or higher were selected and are reported in Table 4. As reported, *FLUOROURACIL*, *EPIRUBICIN,* and *FOSTAMATINIB* are effective drugs and targeted 4, 3, and 3 genes, respectively.

**Table 4:** High degree drugs in the *drug-gene* network.

Drug	Degree	Target genes
FLUOROURACIL	4	BIRC5, GNL3, TYMS, TOP2A
EPIRUBICIN	3	BIRC5, GNL3, TOP2A
FOSTAMATINIB	3	NEK2, CDK1, TTK

Of all the drugs, only those with degree 3 or higher have been reported.

**Figure 4:**
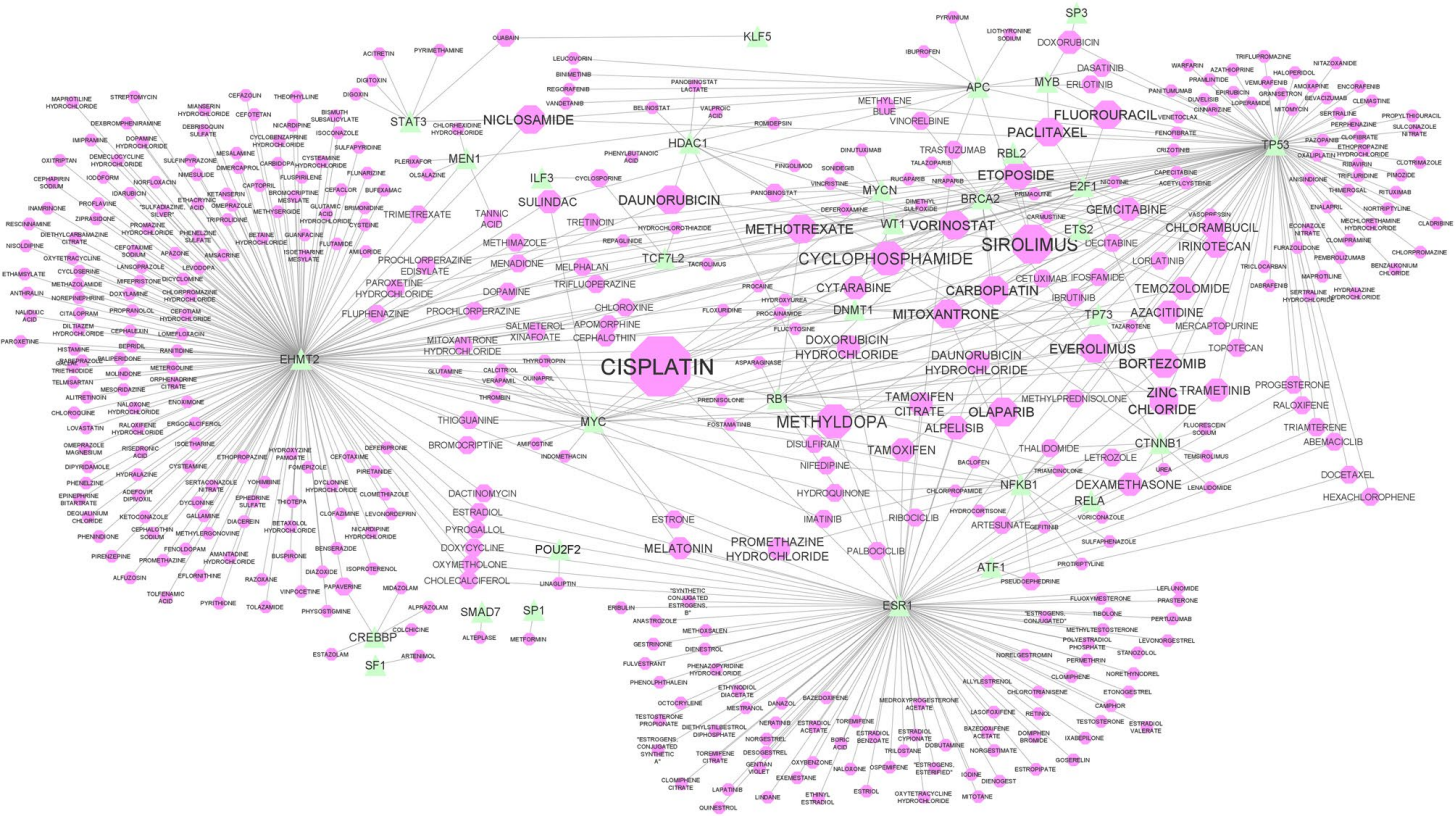
Drug-TF interaction network for *TF-miRNA-TG_A* and *TF-miRNA-TG_B* sub-networks. Green triangle shapes show genes, and pink octagon shapes show drugs. The size of the octagon nodes indicates its degree. The high degree drug is CISPLATIN with 11 target genes.

In addition to the Drug-gene interaction network, the drug-TF network was reconstructed to discover the drugs that target the transcription factors of the *TF-miRNA-TG_A* and *TF-miRNA-TG_B* sub-networks. This network is shown in Fig. 4. In this network, the drugs with a degree of 4 and higher were selected and reported as significant drugs. More details of the effective drugs are reported in Table 5. As the table shows, *CISPLATIN*, *SIROLIMUS,* and *CYCLOPHOSPHAMIDE* drugs targeted 11, 6, and 5 TFS, respectively, and the others targeted 4 TFs. The complete information of Drug-Gene and Drug-TF interactions are provided in supplementary file S5. Additionally, supplemental file S6 has detailed information on the network topological characteristics of the Drug-Gene and Drug-TF networks.

**Table 5:** High degree drugs in the *Drug-TF* network.

Drug	Degree	Target TFs
CISPLATIN	11	BRCA2, DNMT1, E2F1, EHMT2, ESR1, MYC, MYCN, RB1, TP53, TP73
SIROLIMUS	6	APC, RB1, RBL2, TCF7L2, TP53, WT1
CYCLOPHOSPHAMIDE	5	BRCA2, CTNNB1, EHMT2, MYCN, TP53
METHYLDOPA	5	EHMT2, ESR1, HDAC1, TP53, TP73
VORINOSTAT	4	HDAC1, MYC, RB1, TP53
OLAPARIB	4	BRCA2, ESR1, MYC, TP53
MITOXANTRONE	4	DNMT1, EHMT2, NFKB1, TP53
FLUOROURACIL	4	APC, E2F1, MYB, TP53
EVEROLIMUS	4	BRCA2, CTNNB1, ESR1, RB1
PACLITAXEL	4	BRCA2, E2F1, MYB, TP53
DAUNORUBICIN	4	EHMT2, HDAC1, TP53, WT1
ZINC CHLORIDE	4	ESR1, HDAC1, TP53, TP73
METHOTREXATE	4	E2F1, EHMT2, RB1, TP53
CARBOPLATIN	4	BRCA2, ETS2, TP53, TP73
BORTEZOMIB	4	E2F1, NFKB1, RB1, TP53
NICLOSAMIDE	4	APC, EHMT2, STAT3, TP53
ETOPOSIDE	4	BRCA2, E2F1, MYCN, TP53

Of all the drugs, only those with degree 4 or higher have been reported.

Recently, the potential effect of 6710 drugs as SARS-CoV-2 inhibitors is tested in vitro and in vivo^44^. The results of this report show that some of the drugs proposed in this article (see Tables 4 and 5) have an inhibitory effect on SARS-CoV-2. According to this report, *OLAPARIB*, *NICLOSAMID* and *METHOTREXATE* have a *very weak*, *weak*, and *strong* effects on SARS-CoV-2, respectively. As well as, *DAUNORUBICIN* and *BORTEZOMIB* have a cytotoxic effect on this disease.

### GSEA and candidate drugs validation

To evaluate the proposed drugs, the Connectivity Map(CMAP) analysis was utilized. To do this, from the Drug-Gene and Drug-TF networks, the drugs with a degree of 4 or above were selected and imported to Enrichr CMAP database. Then, the impact of these drugs on target genes was evaluated. Enrichr CMAP database contains CMAP-up and CMAP-down datasets. In Tables 3 and 4, from among the 19 repurposed drugs for SARS-CoV disease, only nine drugs, including *SIROLIMUS*, *METHYLDOPA*, *VORINOSTAT*, *PACLITAXEL*, *DAUNORUBICIN*, *METHOTREXATE*, *NICLOSAMIDE,* and *ETOPOSIDE,* were validated by CMAP analysis. Table 6 shows the validated drugs together with the corresponding unregulated or downregulated target genes.

**Table 6:** The validated candidate drugs by CMAP analysis.

Drug name	Gene names (↓:Downregulated and ↑: upregulated)
SIROLIMUS	TYMS (↑), NLE1 (↑), CDC25A (↑), BYSL (**↓**), UTP3 (**↓**), GNL3 (**↓**), MRTO4 (**↓**), UTP6 (**↓**), KRR1 (**↓**), CDC25A (**↓**)
METHYLDOPA	NMD3 (**↓**)
VORINOSTAT	CCNB2 (**↓**)
PACLITAXEL	KHDRBS1 (↑)
DAUNORUBICIN	BUB1B (**↓**), CENPE (**↓**), NMD3 (**↓**)
METHOTREXATE	UBE2C (**↓**), CDC20 (**↓**), BUB1B (**↓**), KIF20A (**↓**), PTTG1 (**↓**), CDKN3 (**↓**), KIF11 (**↓**), TACC3 (**↓**), TOP2A (**↓**), TTK (**↓**), CENPF (**↓**), CCNB2 (**↓**)
NICLOSAMIDE	HNRNPA1 (↑) GINS2 (**↓**), BYSL (**↓**), RRM2 (**↓**)
ETOPOSIDE	UBE2C (**↓**), CDC20 (**↓**), KIF20A (**↓**), NCAPG (**↓**), PTTG1 (**↓**), PNO1 (**↓**), NEK2 (**↓**), CENPF (**↓**), TOP2A (**↓**), KIF11 (**↓**), CDKN3 (**↓**), TACC3 (**↓**), TTK (**↓**)

## Discussion

In this article, a network-based approach was applied to discover therapeutic drugs for SARS-CoV-2 disease. Due to the high genetic similarity of SARS-CoV and SARS-CoV-2, the results of the study on SARS-CoV can be a clue to the treatment of SARS-CoV-2. To this end, at first, differentially expressed significant genes (*P-value* < 0.05) in healthy and SARS-CoV infected samples were selected, then the gene co-expression network was reconstructed in STRING database for the filtered genes.

After reconstructing the gene co-expression network, we discovered two significant gene modules using ClusterViz plugin in Cytoscape software. These two obtained gene co-expression modules (*Module A* and *Module B*) contained 25 and 30 genes, respectively. In the next step, a list of TFs and miRNAs which regulate these module's genes were gathered from the *TRRUST* and *miRWalk2.0* databases, respectively. Moreover, the regulation information of the TFs and miRNAs were obtained from the *TransmiR* database. After collecting the TFs, miRNAs, and TGs regulation information, the two sub-networks of *TF-miRNA-TG_A* and *TF-miRNA-TG_B* were reconstructed. *TF-miRNA-TG_A* contains 347 miRNAs, 25 TGs, and 57 TFs, and *TF-miRNA-TG_B* contains 116 miRNAs, 25 TGs, and 4 TFs.

To analyze the biological processes and pathways in which *Module A* and *Module B* are involved, Gene Ontology (GO) and pathway enrichment analyses were done using a DAVID tool. The GO enrichment analysis showed that the genes in *Module A* significantly enriched in *mitotic cell cycle process, nuclear division,* and* mitotic nuclear division* biological processes. In *Module B*, the genes significantly enriched in *RNA processing*, *mRNA metabolic process,* and *RNA metabolic process* terms*.* The *R*eactome pathway enrichment analysis showed that modules A and *B* enriched in* Resolution of Sister Chromatid Cohesion* and* mRNA Splicing—Major Pathway* terms, respectively (see supplementary file S3)*.* To analyze the *TF-miRNA-TG_A* and *TF-miRNA-TG_B* sub-network's miRNAs, the TAM online tool was utilized. Using this tool, we identified miRNAs families for these sub-networks. The results showed that the miRNAs of both sub-networks significantly enriched in *let-7* and *mir-17* families. Some other significant miRNAs families are reported in the supplementary file S4.

Given that the transcription factors have considerable roles in the gene expression process, the TFs of both *TF-miRNA-TG_A* and *TF-miRNA-TG_B* sub-networks were studied and evaluated. To this end, from any *TF-miRNA-TG* sub-networks, the TFs with a degree of 10 and above were selected and reported. The high-degree TFs were *MYC, TP53, NFKB1, RELA, STAT3, MYCN, SP1, ESR1, E2F3, KLF4, CTNNB1, DNMT1, NANOG, TP73, LEF1, MYB,* and *FOXO3.* These 17 transcription factor genes can be evaluated in further studies on SARS-CoV-2 coronavirus disease.

Hub miRNAs in *TF-miRNA-TG_A* and *TF-miRNA-TG_B* sub-networks are essential and crucial, as they regulate more genes of the subnetworks at a post-translational regulation level and can impact biological processes. Therefore, for each *TF-miRNA-TG* subnetwork, five high degree miRNAs were selected and reported as significant miRNAs. Based on the findings, *hsa-miR-193b, hsa-miR-192, hsa-miR-215, hsa-miR-34a,* and *hsa-miR-16* were significant miRNAs for *TF-miRNA-TG_A* subnetwork, and *hsa-miR-16, hsa-miR-92a, hsa-miR-30a, hsa-miR-7,* and *hsa-miR-26b* were found to be significant miRNAs for *TF-miRNA-TG_B* subnetwork as well. Among these miRNAs*, hsa-miR-16* regulates more genes in both subnetworks. According to the literature, some of these miRNAs have been studied in SARS coronavirus.

Kyung Hee Choi et al.^45^ found that has-miR-193 is a dual-strand tumor suppressor and a novel therapeutic target for lung cancer. In another study, Huajun Hu et al.^46^ reported that this miRNA is a tumor suppressor in Non-small cell lung cancer (NSCLC). Liang Sun et al.^47^ concluded that hsa-miR-193b regulates the RAB22A oncogene, inhibits breast cancer growth, and may have significant implications for cancer therapy. In addition, this miRNA regulates breast cancer cell migration and vasculogenic mimicry by DDAH1^48^. Moreover, it could function as a tumor-suppressive miRNA in breast cancer^49^ and inhibits breast cancer metastasis^50^.

Martyna Filipska et al.^51^ found that hsa-miR-192-5p has a functional role in squamous cell lung cancer cells. In Peng Zou et al.^52^ reported that this miRNA suppresses the progression of lung cancer bone metastasis by targeting TRIM44. Moreover, this miRNA has been introduced as a prognostic marker for NSCLC participants^53^. Importantly, *hsa-miR-192* induces Cisplatin-resistance, inhibits cell apoptosis in lung cancer^54^ and the proliferation, migration, and invasion of osteosarcoma cells, and promotes apoptosis^55^. Xiaopan et al.^56^ reported that *hsa-miR-215* suppresses proliferation and migration of non-small cell lung cancer cells(NSCLC). This miRNA is downregulated in NSCLC tissues and may play a key role in the development of NSCLC. The lower expression of *has-miR-215* in NSCLC is negatively associated with lymphatic metastasis and TNM staging^57^. This miRNA targets ZEB2 in human non-small cell lung cancer and functions as a tumor suppressor^58^. Ariana Centa et al.^59^ expressed that *has-miR-34a-5p* is identified as the regulator of mRNA targets involved in endothelial, inflammatory signaling pathways, and viral diseases. Furthermore, in the present study, the expression of this miRNA was significantly down-regulated in the COVID-19 patients compared to the Controls. Also, Martin Hart et al.^60^, in their systems biology, analysis identified *miR-34a* as strongly associated with pathogenesis. In another study, Rieko Aida et al.^61^ reported that apigenin might induce apoptosis by down-regulating SNAI1 through *miR-34a-5p* up-regulation in A549 cells. Woo Ryung Kim et al.^62^ reported that *hsa-miR-16-5p* is commonly bound to SARS-CoV, MERS-CoV, and SARS-CoV-2. In Zofia Wicik et al.^63^ showed that this miRNA could regulate ACE2 networks. Moreover, this miRNA can link the pathogenesis of HIV-1 and malaria^64–66^. Similarly, Jianghong Wei et al.^67^ found that overexpression of miR-16 inhibited the growth and metastasis of the DMS-53 lung cancer cells. Alireza Paniri et al.^68^ reported that *hsa-miR-26b-5p* strongly targets *ACE2 and have an* important effect on SARS coronavirus. Like has-miR-16-5p, has-mir-26b-5p can regulate ACE2 networks as well^63^. Moreover, this paper reported that has-miR-26b-5p may plays a significant role in the pathogenesis of HF in COVID-19 patients. The effect of this miRNA in SARS coronavirus was studied by Laura Teodori and her colleagues^69^. Moreover, Yang Gao et al.^70^ reported that this miRNA plays an important role in tumor suppression in lung cancer. According to M Xia et al., this miRNA could suppress lung cancer cells' proliferation, migration, and invasion. Min Jiang et al.^71^ reported that *has-miR-92a* family could be ideal biomarkers for cancer diagnosis and prognosis. Also, our study revealed that the expression of *has-mir-92a* was upregulated in lung squamous cell carcinoma (LUSC). Besides, this miRNA could promote growth, metastasis, and chemoresistance in NSCLC cells^72^. This miRNA was thus introduced as a plasma biomarker for small cell lung cancer^73^. Jianhua Gong et al.^74^ revealed that the *has-miR-92a* up-regulation could significantly induce proliferation and inhibit apoptosis of lung cancer cells. Jianjie Zhu et al.^75^ revealed that the upregulation of *has-mir-30-5p* in lung cancer cell lines inhibited cell proliferation in vitro and in vivo. This miRNA suppresses lung cancer progression by targeting SIRT1^76^. Also, the lack of its expression promotes the growth of lung cancer cells by targeting MEF2D. Moreover, Xiaowei Quan et al.^77^ and Ruixue Tang et al.^78^ revealed that miR-30a-5p expression is downregulated in NSCLC. In addition, the increase in miR-30a-5p level could enhance Bax protein level and decrease Bcl-2 protein level^77^. In the field of pharmaceutical research, Xiaojie Xu et al.^79^ reported that miR-30a-5p enhances paclitaxel sensitivity in non-small cell lung cancer through targeting BCL-2 expression. Haiping Xiao et al.^80^ believed that *has-miR-7-5p* suppresses tumor metastasis of NSCLC by targeting NOVA2. Plus, Kenneth Lundstrom^81^ revealed that Rotavirus (RV) miR-7 can inhibit rotavirus replication by targeting the RV nonstructural protein 5. In another study, it was found that has-miR-7 could repress fibrogenesis of lung fibroblasts induced by TGF-β1^82^. In addition, Xiaofei Zhang et al.^83^ reported that the overexpressed CDR1as functions as an oncogene to promote the tumor progression via miR-7 in non-small-cell lung cancer.

From the perspective of pharmacological studies, our finding shows that 470 drugs target TF and non-TF genes in both TF-miRNA-TG_A and TF-miRNA-TG_B subnetworks. Of 470 drugs, 62 drugs target both TF and non-TF genes, 95 drugs target non-TF genes, and 436 drugs target TF genes. From among the 470 obtained drugs, the drugs which target more genes were selected and discussed. In the drug-gene network, the drugs with a degree of 3 or above were selected and reported as potential and effective drugs for treating patients infected with SARS coronavirus. These drugs, including *FLUOROURACIL*, *EPIRUBICIN,* and *FOSTAMATINIB,* target at least three genes in the drug-gene network. Also, the drugs with a degree of 4 or above were selected and reported for the drug-TF network. These high-degree drugs were *CISPLATIN, SIROLIMUS, CYCLOPHOSPHAMIDE, METHYLDOPA, VORINOSTAT, OLAPARIB, MITOXANTRONE, FLUOROURACIL, EVEROLIMUS, PACLITAXEL, DAUNORUBICIN, ZINC CHLORIDE, METHOTREXATE, CARBOPLATIN, BORTEZOMIB, NICLOSAMIDE,* and *ETOPOSIDE.* Of the reported drugs, *CISPLATIN* targets 11 Transcription factor genes and may have a crucial impact on SARS coronavirus disease. We found that some of these drugs have been studied in SARS-CoV and SARS-CoV-2 coronaviruses, and others can be assumed as candidate drugs for SARS-CoV-2 coronavirus disease therapeutic.

Shamim I. Ahmad, in his recent study, revealed that *FLUOROURACIL,* in combination with deoxyribose and deoxyribonucleosides, can be a therapeutic option for SARS coronavirus^84^. *EPIRUBICIN*, VAPREOTIDA, and SAQUINAVIR have been proposed as key drugs in SARS coronavirus treatment^85^. Also, Strich et al.^86^ introduced the *FOSTAMATINIB* as a potential therapeutic for COVID-19. Moreover, *FOSTAMATINIB* has the potential to treat serious outcomes of coronavirus COVID-19, including acute lung injury (ALI) and acute respiratory distress syndrome (ARDS)^87,88^. In addition, several studies evaluated and showed the impact of *FOSTAMATINIB* on SARS coronavirus^89–92^. The mTOR signaling plays a crucial role in MERS-CoV infection^93^. In this regard, Yadi Zhou et al.^34^ observed that the *SIROLIMUS* is an inhibitor of mTOR with both antifungal and antineoplastic properties. In addition, this drug has been presented as a viral protein expression blocker^94^. Swaroop Revannasiddaiaha et al.^95^ showed that *CYCLOPHOSPHAMIDE* had a potential role in mitigation of acute respiratory distress syndrome among patients with SARS-CoV-2. Moreover, Brocato et al.^96^ , Othenin-Girard et al.^97^, Corso et al.^98^, Schaecher et al.^99^, and Revannasiddaiah et al.^95^ evaluated and showed the impact of *CYCLOPHOSPHAMIDE* on SARS coronavirus disease with different approaches.

Al-Rashedi et al.^100^ noted that the *OLAPARIB* is a potential drug for treating patients infected with SARS-COV-2. *MITOXANTRONE* has also been introduced as potential inhibitors of SARS-CoV-2 M^pro^^101^. Safavi et al.^102^ showed that the *METHOTREXATE* silence the immune activation in patients with COVID-19. Also, Sujoy Khan et al.^103^ proposed this drug as a potential drug for treating patients infected with COVID-19. Additionally, this drug has a protective effect on SARS-CoV-2 infection via downregulating ACE2^104^. In this study, another drug that we have reported as a potential drug for SARS coronavirus was *NICLOSAMIDE*. This drug has previously been reported as an antiviral agent against COVID-19^105,106^. Other studies have reported the potential of this drug in treating patients infected with COVID-19^107,108^. Different studies have been undertaken on the effect of *ETOPOSIDE* on SARS coronavirus disease^109–112^. In our previous research based on protein–protein-network analysis, we proposed *PACLITAXEL*, *CARBOPLATIN*, *BORTEZOMIB*, *VORINOSTAT,* and *DAUNORUBICIN* as potential drugs for SARS-CoV-2 coronavirus treatment^21^. In this study, *PACLITAXEL* was introduced as the most potent therapeutic candidate drug. In previous research, rare studies examined the effect of this drug on SARS-CoV-2 disease.

In conclusion, based on our results, these 19 drugs can be assumed as candidate therapeutic drugs for SARS-CoV-2 coronavirus. Moreover, along with some other drugs, nine miRNAs were proposed as candidate miRNAs, which may play an important role in treating SARS-CoV-2 disease. These candidate miRNAs include *hsa-miR-193b, hsa-miR-192, hsa-miR-215, hsa-miR-34a,hsa-miR-16, hsa-miR-92a, hsa-miR-30a, hsa-miR-7,* and *hsa-miR-26b*.

## Conclusion

In this study, focusing on the gene expression profile of SARS-CoV samples, an attempt was made to identify effective drugs for the treatment of this disease with a gene co-expression network-based approach. Given that the genomes of SARS-CoV and SARS-CoV-2 are very similar, it is expected that the drugs introduced to treat SARS-CoV coronavirus would also be effective in treating SARS-CoV-2 disease. Current research aimed to discover novel potential drugs for SARS-CoV disease in order to treating SARS-CoV-2 coronavirus based on a co-expression network analysis. To this end, at first, significant DEGs in normal and SARS-CoV infected samples were selected and then the gene co-expression network was reconstructed and two gene modules were discovered as significant modules. Then, two significant gene modules were discovered from the reconstructed co-expression network. Next, for the obtained modules, two sub-networks named *TF-miRNA-TG_A* and *TF-miRNA-TG_B* were drawn. Afterward, the list of the drugs targeting *TF-miRNA-TG_A* and *TF-miRNA-TG_B* sub-networks' genes was extracted, and two drug-gene and drug-TF interaction networks were drawn. Eventually, five drugs including *FLUOROURACIL*, *CISPLATIN*, *SIROLIMUS*, *CYCLOPHOSPHAMIDE*, and *METHYLDOPA* are proposed as poteintial drugs for SARS-CoV-2 coronavirus treatment. As well as, ten miRNAs including* miR-193b, miR-192, miR-215, miR-34a, miR-16, miR-16, miR-92a, miR-30a, miR-7,* and *miR-26b* were found to be significant miRNAs in treating SARS-CoV-2 coronavirus.

## Methods

### Dataset and preprocessing

The gene expression data used in this work were downloaded from the NCBI Gene Expression Omnibus (GEO) database with the accession number GSE1739. This data contains gene expression profiles of normal and Severe Acute Respiratory Syndrome (SARS) infected patients' blood samples. To assign probes to gene IDs, the annotation file published by Affymetrix was used.

In this article, a network-based approach was applied to discover therapeutic drugs for SARS-CoV-2 disease. To this end, at first, differentially expressed significant genes (*p_value* < *0.05*) in healthy and SARS-CoV infected samples were selected, and then the gene co-expression network was reconstructed in STRING database for the filtered genes.

### Network reconstruction and module extraction

At first, differentially expressed genes were extracted for the normal and SARS infected groups. In order to calculate the differentially expressed genes, adjusted p_value was calculated using *Benjamini & Hochberg false discovery rate* method. Then, 1441 genes with *p-values* less than 0.05 were assumed as primary genes, which were then used in network reconstruction. The list of mentioned primary genes is reported in Supplementary file S1. Afterward, the gene co-expression network is reconstructed by primary genes in STRING database. In this web tool, the minimum required interaction score parameter is adjusted to 0.04.

In order to analyze the reconstructed co-expression network, the Cytoscape software^43^ version 3.8.2 was used. ClusterViz^113^ plugin was used to identify gene modules (highly interconnected regions) in the co-expression network. ClusterViz is a Cytoscpae plugin, which discovers modules in a biological network using various clustering algorithms. This plugin contains three commonly used clustering algorithms, including FAG-EC, EAGLE, and MCODE.

In this research, we have applied all of the algorithms and the results did not have significant difference, so we have decided to select one of them. Therefore, the MCODE (Molecular Complex Detection) algorithm was used to find the gene co-expression modules. MCODE is a graph theoretic clustering algorithm for discovering strongly connected regions in a given network^113^. This algorithm selects the seed nodes and expand them based on the density of the cluster and density of the local neighborhood^113^. The MCODE algorithm was performed with the following parameters: Degree threshold = 2, NodeScore Threshold = 0.2, K-Core Threshold = 2, and Maxdepth = 100.

### Transcription factors, miRNAs, and target genes interaction network

To investigate the effect of Transcription Factors (TF) and microRNAs (miRNA) on target genes (TG), the *TF-miRNA-TG* sub-networks were reconstructed for gene modules.

Transcription factors (TF) are proteins that regulate the rate of transcription of genetic information from DNA to messenger RNA^114^. miRNAs are small non-coding RNAs that function in RNA silencing and post-transcriptional gene regulation^23,115^. Both TFs and miRNAs regulate gene expression^116^. To get regulatory interactions information of TFs-TGs, the TRRUST online database was utilized. This database contains 8,444 and 6,552 TF-target regulatory relationships of 800 human TFs and 828 mouse TFs, respectively. In addition to TFs-TGs regulatory information, TFs-miRNAs regulatory interactions are essential. This information is obtained from the TransmiR V2^42^ database. TransmiR v2.0 incorporates 3,730 TF-miRNA regulatory interactions, covering 623 TFs and 785 miRNAs for 19 organisms. In this study, the information of miRNAs-TGs regulatory interactions was retrieved from the miRWalk 2.0 database. This database contains both validated and predicted interactions. In this study, only experimentally validated miRNAs–TGs interactions were considered.

In order to analyze the reconstructed co-expression network, the Cytoscape software was used. After analyzing the network, generally, 391 nodes and 1273 edges were observed in the reconstructed co-expression network. ClusterViz^113^ plugin was used to identify gene modules (highly interconnected regions) in the co-expression network. ClusterViz is a Cytoscpae plugin, which discovers modules in a biological network using various clustering algorithms. This plugin contains three commonly used clustering algorithms, including FAG-EC, EAGLE, and MCODE. The MCODE (Molecular Complex Detection) algorithm was used to find the gene co-expression modules in this study. The MCODE algorithm was performed with the following parameters: Degree threshold = 2, NodeScore Threshold = 0.2, K-Core Threshold = 2, and Maxdepth = 100. After clustering the gene co-expression network, we discovered two significant modules (Module A and Module B) in the co-expression network.

### Enrichment analysis

The Database for Annotation, Visualization, and Integrated Discovery (DAVID ) v6.8^117,118^ was used for the enrichment analysis of the genes. The gene ontology and pathway enrichment analysis were done for obtained gene modules.

To identify miRNAs family, the TAM online tool^119^ was applied. To do this, all the miRNAs in *TF-miRNA-TG* sub-networks were imported to the TAM tool separately, and then significant miRNA families were identified.

### Drug-Gene interaction network

To identify the drugs that target *TF-miRNA-TG_A* and *TF-miRNA-TG_B* TF and non-TF genes, the Drug Gene Interaction Database (DGIdb)^41^ was used. This database retrieves drug-gene interaction information from 24 other related databases. In this study, to identify drug-genes interaction, only approved drugs were used.

### GSEA and candidate drugs validation

In order to evaluate the proposed drugs for SARS-CoV-2 disease, the GSEA was performed by querying the Enrichr database^120^. To this end, the Enrichr database was utilized to perform the Connectivity Map(CMAP) analysis^121^.

## Supplementary Information

Supplementary Information 1.

Supplementary Information 2.

Supplementary Information 3.

Supplementary Information 4.

Supplementary Information 5.

Supplementary Information 6.

Supplementary Information 7.
