# Supplementary material for: Drug repurposing for coronavirus (SARS-CoV-2) based on gene co-expression network analysis
Source: Sci Rep. 2021 Nov 8;11:21872. doi: 10.1038/s41598-021-01410-3 (PMC8576023; doi:10.1038/s41598-021-01410-3)
Supplement: Supplementary file 7 — Supplementary Information 7. [file 41598_2021_1410_MOESM7_ESM.docx]

**Drug repurposing for coronavirus (SARS-CoV-2) based on gene co-expression network analysis**

Habib MotieGhader PhD, Esmaeil Safavi PhD, Ali Rezapour PhD, Fatemeh Firouzi Amoodizaj MSc, Roya asl iranifam MSc

Figure S1. *TF-miRNA-TG_A* and *TF-miRNA-TG_B* subnetworks*.* Green triangle, shapes show TFs, Blue circle shapes show TGs, and red diamond shapes show miRNAs*.* *TF-miRNA-TG_A* contains 4 Transcription factors, 25 genes, and 116 miRNAs. *As well as, TF-miRNA-TG_B* contains 56 transcription factors, 26 genes, and 429 miRNAs. The comprehensive topological properties of these sub-networks are reported in Supplementary file S6*.*

| *TF-miRNA-TG_A*  **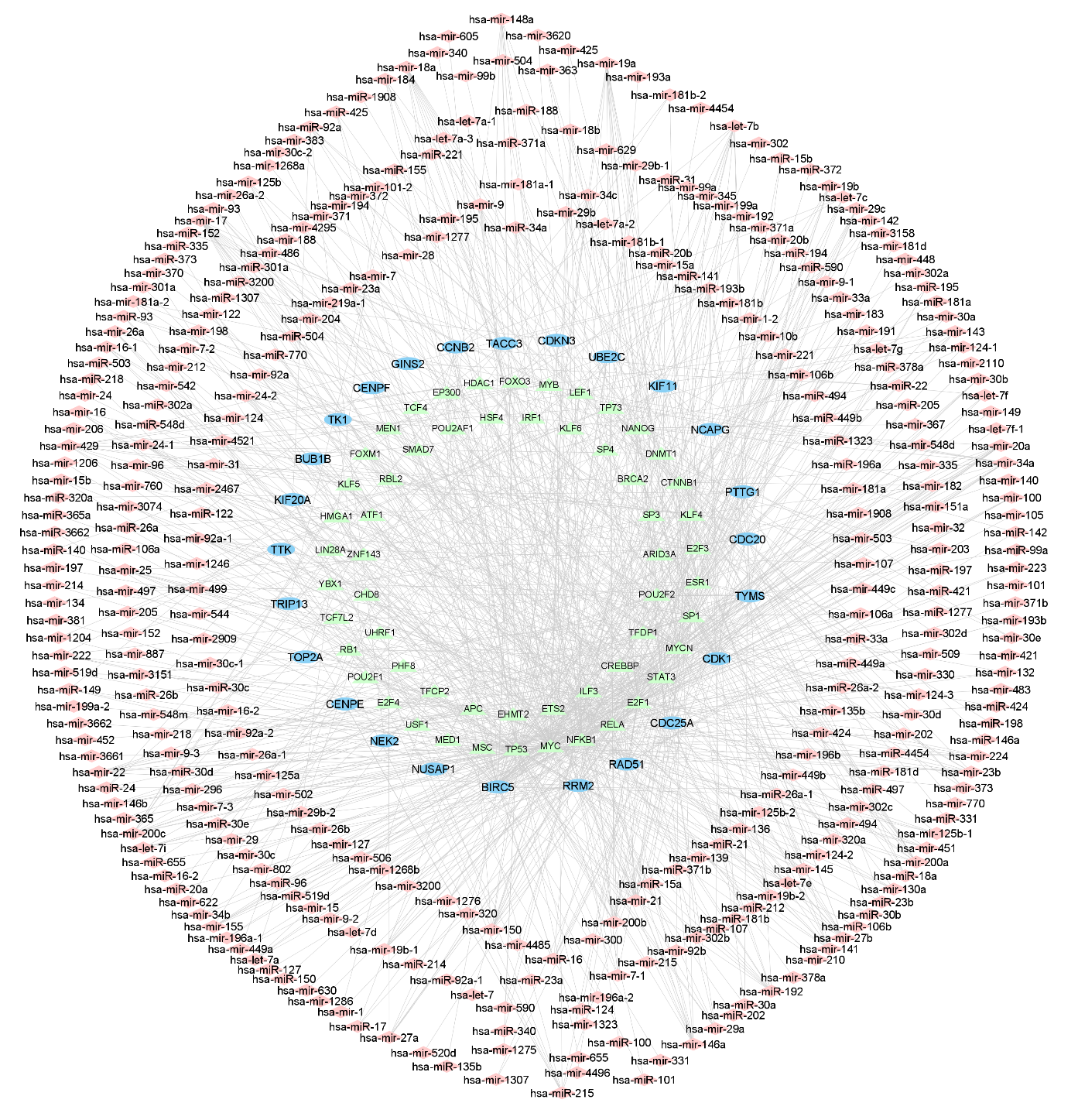** |
| --- |
| *TF-miRNA-TG_B*  **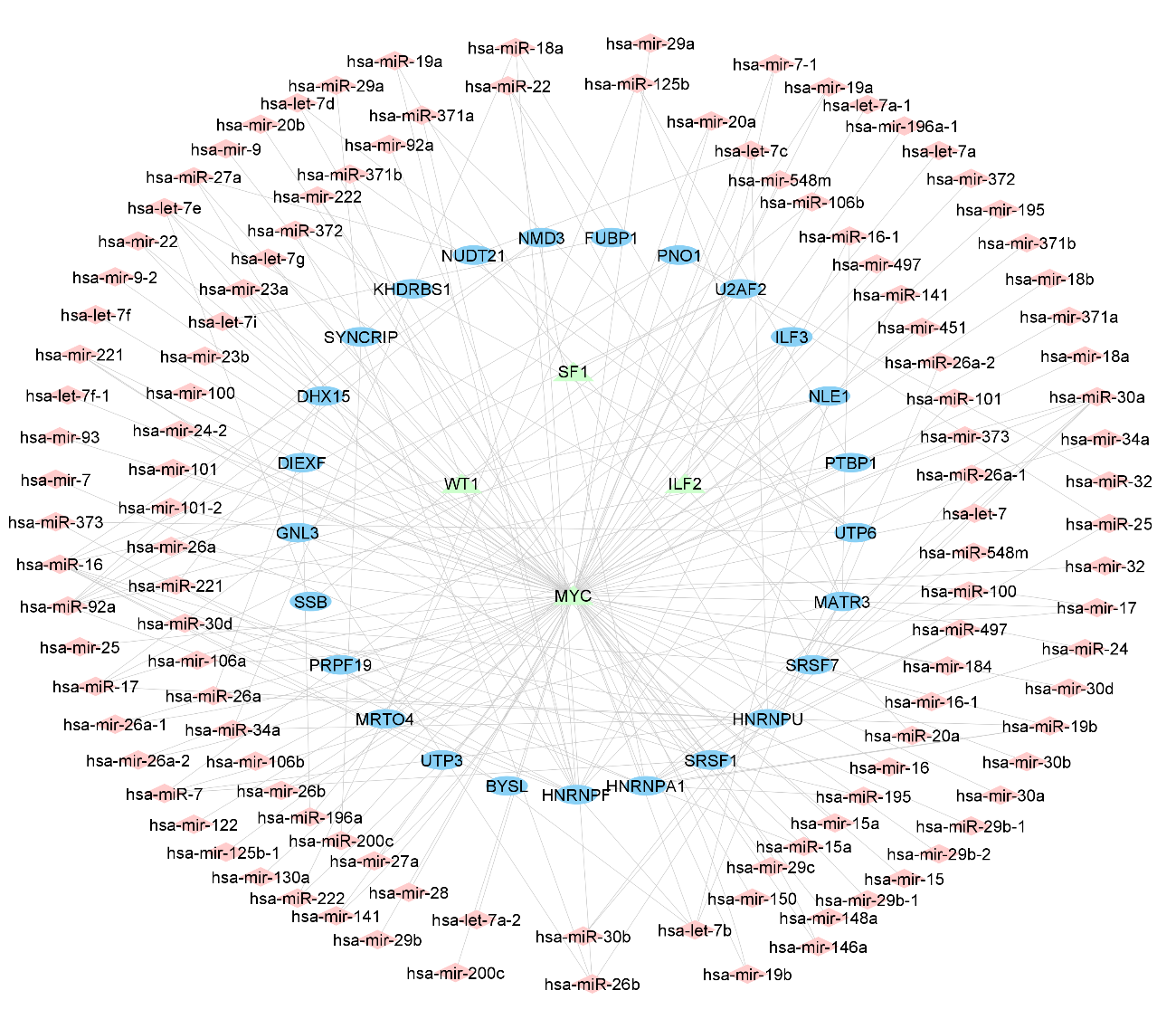** |
